# Artificial Intelligence Empowers Novice Users to Acquire Diagnostic-Quality Echocardiography

**DOI:** 10.1016/j.jacadv.2025.102005

**Published:** 2025-07-22

**Authors:** Biana Trost, Laetitia Rodrigues, Caroline Ong, Alexandre Dezellus, Ythan H. Goldberg, Marine Bouchat, Emilie Roger, Olivier Moal, Varinder Singh, Bertrand Moal, Stephane Lafitte

**Affiliations:** a Northwell, New Hyde Park, New York, USA; b Department of Cardiology, Lenox Hill Hospital, New York, USA; c DESKi, Bordeaux, France; d Department of Cardiology, Nouvelle Clinique Bordeaux Tondu, Floirac, France; e Department of Cardiac and Vascular Surgery, Centre Hospitalier Universitaire de Bordeaux, Hopital Cardiologique, Pessac, France

**Keywords:** artificial intelligence, cardiac ultrasound, deep learning, echocardiography

## Abstract

**Background:**

Cardiac ultrasound exams provide real-time data to guide clinical decisions but require highly trained sonographers. Artificial intelligence (AI) that uses deep learning algorithms to guide novices in the acquisition of diagnostic echocardiographic studies may broaden access and improve care.

**Objectives:**

The objective of this trial was to evaluate whether nurses without previous ultrasound experience (novices) could obtain diagnostic-quality acquisitions of 10 echocardiographic views using AI-based software.

**Methods:**

This noninferiority study was prospective, international, nonrandomized, and conducted at 2 medical centers, in the United States and France, from November 2023 to August 2024. Two limited cardiac exams were performed on adult patients scheduled for a clinically indicated echocardiogram; one was conducted by a novice using AI guidance and one by an expert (experienced sonographer or cardiologist) without it. Primary endpoints were evaluated by 5 experienced cardiologists to assess whether the novice exam was of sufficient quality to visually analyze the left ventricular size and function, the right ventricle size, and the presence of nontrivial pericardial effusion. Secondary endpoints included 8 additional cardiac parameters.

**Results:**

A total of 240 patients (mean age 62.6 years; 117 women (48.8%); mean body mass index 26.6 kg/m^2^) completed the study. One hundred percent of the exams performed by novices with the studied software were of sufficient quality to assess the primary endpoints. Cardiac parameters assessed in exams conducted by novices and experts were strongly correlated.

**Conclusions:**

AI-based software provides a safe means for novices to perform diagnostic-quality cardiac ultrasounds after a short training period.

Heart disease is the world's leading cause of death, with 20 million fatalities globally in 2021 and over half a billion people affected worldwide.^1^ Echocardiography is a key diagnostic modality and is often the first step in cardiac care due to its noninvasive, portable, and affordable nature. Beyond its use by cardiologists, echocardiography has been successfully used by other medical specialties, particularly in intensive care units for hemodynamic failure or in intra- and extra-hospital emergency medicine for the initial assessment of chest pain or dyspnea.^2^, ^3^, ^4^, ^5^

The miniaturization and price reduction of ultrasound systems have accelerated the expansion of echocardiography. Recently, probes that directly connect to tablets or smartphones have been developed at a relatively low cost. These ultrasound probes can now be considered the “new stethoscope,” potentially used by any health care professional (primary care physicians, nurses, emergency responders, etc) to provide timely clinical care.^6^, ^7^, ^8^

However, a significant limitation to the widespread use of this technology is the complexity of obtaining standardized views for nonspecialists (ie, noncardiologists) with limited expertise in the field. Diagnostic echocardiograms are typically performed by trained cardiac sonographers and interpreted by expert cardiologists. Performing an echocardiogram examination requires anatomical knowledge and practice,^9^ as it involves viewing the heart from different standardized points on the chest.

The operator must first learn to handle the probe and be able to obtain these key reference views (parasternal long axis, parasternal short axis, and subcostal views).

The patient's morphology, the shape of the thorax, the exact position of the heart, the movements of the heart according to the position of the patient, the position of the ribs, and breathing movements are all elements to be considered. These factors make each examination unique.

In the United States today, there are approximately 40,000 cardiologists,^10^ 90,000 sonographers,^11^ and 50,000 emergency doctors.^12^ Additionally, primary care physicians,^13^ nurse practitioners, and registered nurses—totaling over 5 million U.S. professionals—could facilitate the front-line use of echocardiography.^10^ For the past 5 years, companies specializing in deep learning and medical imaging have been developing artificial intelligence (AI)-based software aiming at guiding novices in acquiring cardiac ultrasound exams. These studied AI-driven solutions^14^^,^^15^ can provide real-time feedback to the operator, indicating the movements to perform to obtain the required reference views.

The objective of this prospective trial (NCT05874128) was to evaluate new AI-based software providing real-time guidance during echocardiography for novices to obtain diagnostic-quality acquisitions of 10 standard echocardiographic views.

## Methods

### Selection and description of participants

**Figure 1:**
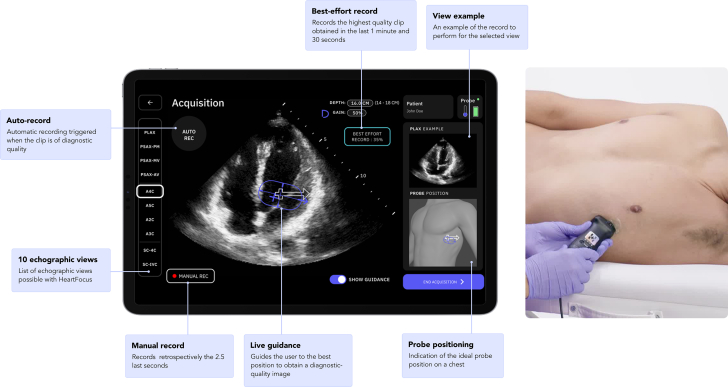
Artificial Intelligence-Based Software User Interface and Setup for Echocardiographic Acquisition An example of the studied application screen (left) showing the actual view in real-time with the Live Guidance items (middle of the screen), the recording features (auto record, best-effort record, manual record), the 10 echocardiographic views (left of the screen), the view example and the probe positioning (right of the screen), and the setup for the apical 4-chamber view acquisition. A2C = apical 2-chamber; A3C = apical 3-chamber; A4C = apical 4-chamber; A5C = apical 5-chamber; PLAX = parasternal long-axis; PSAX-AV = parasternal short-axis at the aortic valve; PSAX-MV = parasternal short-axis at the mitral valve; PSAX-PM = parasternal short-axis at the papillary muscle; SC-4C = subcostal 4-chamber; SC-IVC = subcostal inferior vena cava.

This prospective, multicentric, international, nonrandomized, noninferiority pivotal investigation evaluated new AI-based software (HeartFocus, DESKi (Figure 1). This clinical investigation took place at the University Hospital of Bordeaux in France and Lenox Hill Hospital in New York, United States, from November 2023 to August 2024. The study was approved by an Ethics Committee for the French investigation center and by an Institutional Review Board for the American investigation center. It was conducted following the ethical principles of the Helsinki Declaration, and written consent was obtained from each participant.

The main hypothesis for the sample size calculation of this pivotal trial was based on the sequential testing of the 4 primary endpoints (left ventricular [LV] size, LV function, size of the right ventricle [RV], and presence of nontrivial pericardial effusion [PE]) with the performance goal of 80% (each lower 2-sided 95% CI limit for each parameter should be >80%). Thus, the sample size (240 patients and 8 different novice operators who performed the acquisitions on 30 patients) has a statistical power of 0.98 for each parameter or >0.92 for 4 sequentially tested parameters.

Adults aged 18 years and older who were scheduled for a clinically indicated echocardiography examination at one of the 2 investigation centers were consecutively included in this study between November 2023 and August 2024. Individuals were only excluded if they had known anatomical conditions limiting the acquisition of one or more reference views (including known chest deformity and pneumonectomy) or if they were unable to provide informed consent. Patients' body mass indexes (BMIs) and prior cardiac diagnoses were collected from each center's clinical records. Ethnicity and race were collected only at the American site, as French law prohibits the collection of such data. The study population consisted of a representative sample of inpatients and outpatients undergoing echocardiographic examinations in such large hospitals.

### Data collection

This clinical trial evaluated the ability of the studied software to provide live guidance, diagnostic-quality view detection, and auto recordings to assist novices in the acquisition of cardiac ultrasound images (Central Illustration).

**Central Illustration:**
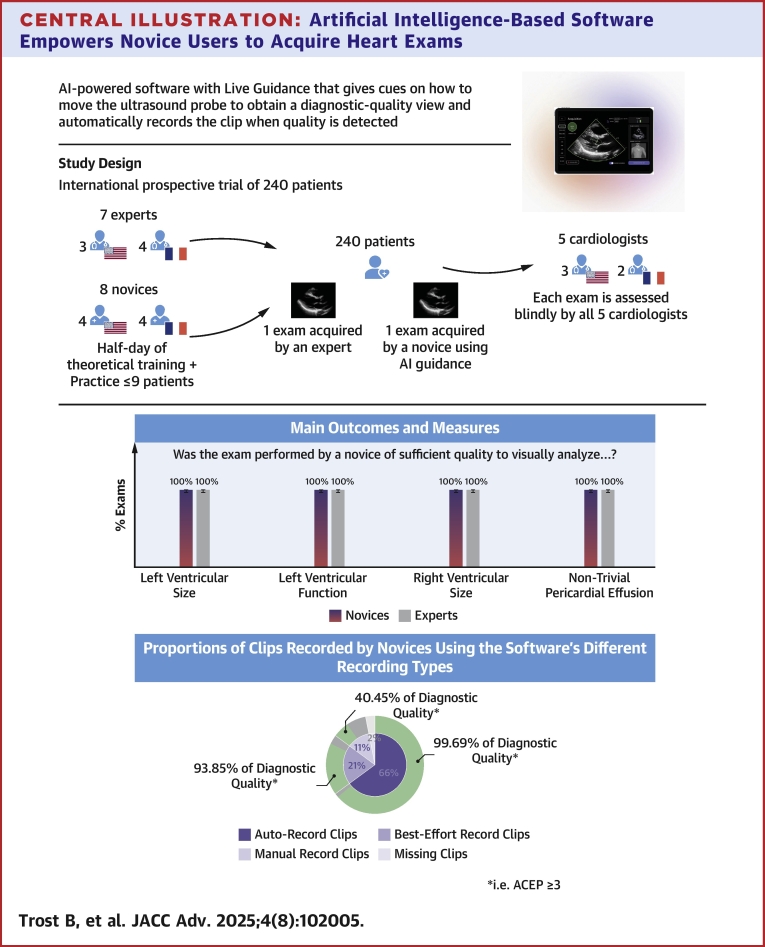
Artificial Intelligence-Based Software Empowers Novice Users to Acquire Heart Exams The AI-powered software provides real-time guidance to assist novices in acquiring diagnostic-quality echocardiographic views, with automated clip recording. The study included 240 patients who underwent 2 echocardiography exams: one by an expert and one by a novice using AI guidance. All exams were independently assessed by 5 cardiologists blinded to the operator type. Primary endpoints included the assessment of left ventricular size and function, right ventricular size, and nontrivial pericardial effusion. Results demonstrated no significant difference in assessment between novice and expert exams. Diagnostic quality was achieved by the novices on 99.7% of the clips recorded with the “auto record” feature, 93.9% of the clips recorded with the “best-effort record” feature, and 40.5% of the clips recorded with the “manual record.” This clinical trial demonstrated that novices can perform heart exams, allowing the evaluation of the most important cardiac parameters. AI = artificial intelligence.

The AI-based software evaluated in this investigation, HeartFocus (DESKi) (see Figure 1), is a computer-assisted acquisition guidance system that provides real-time user guidance during echocardiography, assisting the user in the acquisition of anatomically standard diagnostic-quality 2D echocardiographic views. It supports the acquisitions of 10 echocardiographic views: parasternal long-axis, parasternal short-axis at the aortic valve, parasternal short-axis at the mitral valve, parasternal short-axis at the papillary muscle, apical 4-chamber, apical 5-chamber, apical 2-chamber, apical 3-chamber, subcostal 4-chamber, and subcostal inferior vena cava (IVC). HeartFocus connects to an ultrasound system to receive a stream of ultrasound images; in this clinical trial, the studied software was used with the ultrasound phased array HD3 probe (Clarius).

The studied device proposes several functionalities to assist health care professionals in the acquisition of cardiac ultrasound images. The live guidance feature provides real-time cues to the user on how to move the probe on the patient's chest to obtain diagnostic-quality images. Once the software detects anatomical landmarks, the Live Guidance is displayed with an oval representing the current position of the probe superimposed on a static white arrow indicating the target position. When the probe is optimally positioned and the image reaches diagnostic quality, the user holds the probe in position to enable automated recording. Additionally, the device continually assesses clip quality during scanning; if a suitable clip for auto record cannot be captured, the software allows the user to retrospectively record the highest quality clip obtained so far, referred to as best-effort record.

Patients included in this study received 2 limited ultrasound exams, each consisting of the acquisition of ultrasound clips for each of the 10 echocardiographic views covered by the software. One exam was acquired by a novice using the studied software with its guidance system, while the other was performed by an expert without the assistance of the guidance system. Novices, who were nurses without prior ultrasound experience, received a half-day training on the basics of cardiac ultrasound and on the use of the AI-based software. Each novice practiced on up to 9 patients before starting the study. Eight novices, 4 at each center, completed acquisitions on 30 different patients each. At the French site, 4 expert cardiologists with an echocardiography fellowship, more than 5 years of cardiac ultrasound practice, and experience acquiring more than 30 cardiac ultrasound exams per week acquired the exams on 120 patients. The American site had 3 experts for the 120 patients included, either Registered Cardiac Sonographers through Cardiovascular Credentialing International or Registered Diagnostic Cardiac Sonographers through American Registry of Diagnostic Medical Sonographers, with a diversity of experience in adult echocardiography of 12 to 36 years.

### Measurements

Each limited exam was then assessed by a review committee composed of 5 expert cardiologists (3 from the United States and 2 from France). These cardiologists were all blinded to whether the exam was performed by a novice or an expert, as well as the center where the exam took place. The American cardiologists were certified by the National Board of Echocardiography (level 3 training), while the French cardiologists had a University Diploma in Cardiac Ultrasound, all interpreting more than 30 cardiac ultrasound exams per week. Each cardiologist reviewed each exam. Importantly, none of the cardiologists were among the experts who performed exams during this trial.

The review committee of cardiologists assessed 4 primary endpoints to determine if the ultrasound exams performed by novices were of sufficient quality to visually analyze the LV size, the LV function, the RV size, and the presence of nontrivial PE. This evaluation also included 8 additional secondary endpoints consisting of the function of the RV, the size of the IVC, the size of the left atrium, the size of the right atrium, the aortic valve, the mitral valve, the tricuspid valve, and the segmental kinetics. The cardiologists further evaluated if the quality of each clip was sufficient for clinical interpretation (ie, ratings of American College of Emergency Physicians^16^ grade ≥3 out of 5 were considered “diagnostic quality”) and performed a qualitative and quantitative assessment of the ultrasound measurements. Note that if at least 3 out of 5 reviewers assigned a diagnostic score, the clip was considered of acceptable quality. The correlation between measurements obtained from novice-acquired and expert-acquired studies was systematically analyzed. Then, they visually determined the presence of LV or RV hypertrophy, dilation of the left or RV or atrium, abnormal LV or RV function, abnormal mitral or tricuspid or aortic valve (ie, structurally normal, abnormal, or suspected device), PE, dilation of the IVC, or any other abnormality.

The cardiologists also measured the LV end-systolic and end-diastolic volumes and function. On parasternal analysis, they measured the septal and posterior wall thickness, the internal diameter of the LV (systole and diastole), the aortic root, and the diameter of the IVC. The acquisition time for the limited ultrasound exam for the novices was also collected.

### Statistics

Statistical analyses were carried out by a qualified biostatistician using SAS software (V9.4 or later), R (CRAN), or specific software for multireader multicase study analysis. All patients from the eligible population who underwent the 2 limited exams are included in the full analysis set population, used for the analyses of primary and secondary endpoints. The full analysis set population is described in terms of demographics, ultrasound parameters, and pathology. For all quality criteria, the percentage of success and its CI were evaluated for both expert and novice acquisitions. This analysis was performed using a multireader, multicase power analysis.^17^, ^18^, ^19^ For each qualitative ultrasound measurement, with the expert acquisition serving as the reference, several parameters were evaluated: percentage of agreement, sensitivity, specificity, precision, recall, and F1-score. For each quantitative ultrasound measurement, the differences between measurements derived from the expert acquisitions and those from the novice acquisitions were expressed as mean, SD, and 95% CIs using the Wilson method. For the endpoints, agreement percentages were reported when all 3 cardiologists agreed, as well as when only 2 agreed. Additionally, an intraobserver and an interobserver study were conducted to assess the reproducibility of a review by the same cardiologist or by 2 different cardiologists. Sensitivity studies were performed on the investigating centers, prior cardiac diagnoses, and obesity status.

## Results

**Figure 2:**
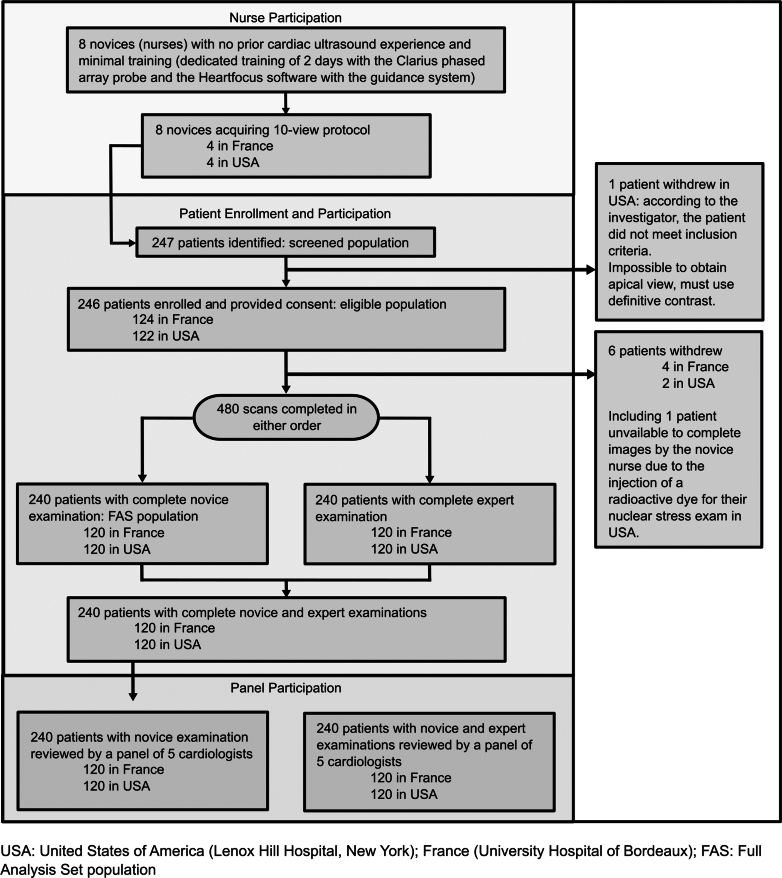
Patient Flowchart The flowchart includes nurse participation, patient enrollment and participation (reasons for withdrawal are specified), and panel participation.

As shown in Figure 2, out of the 247 patients screened, 246 were enrolled and provided consent; of these, 6 withdrew from the study before completing exams. A total of 240 patients (mean age 62.6 ± 15.1 years; 117 women [48.8%]; mean body mass index: 26.6 kg/m^2^) completed the study, with equal distribution across the 2 investigation centers. Ninety-seven patients (40.4%) had a BMI <25, 93 (38.8%) had a BMI between 25 and 30 (overweight), and 50 (20.8%) had a BMI ≥30 (obesity) (see Table 1). Among the studied cohort, 169 patients (70.4%) had known cardiac abnormalities, including 44 (18.3%) with implanted cardiac devices (see Supplemental Table 1).

**Table 1:** Demographic Data: FAS Population (N = 240)

Demographic Variable	Study Site		All (N = 240)
	France (n = 120)	United States (n = 120)	
Sex, n (%)			
Female	47 (39.2)	70 (58.3)	117 (48.8)
Male	73 (60.8)	50 (41.7)	123 (51.2)
Ethnicity, n (%)			
Hispanic or Latino	-	18 (15.0)	-
Not Hispanic or Latino	-	90 (75.0)	-
Unknown/not reported	-	12 (10.0)	-
Race, n (%)			
White	-	52 (43.3)	-
Black/African American	-	36 (30.0)	-
Asian	-	3 (2.5)	-
American Indian/Alaska Native	-	0 (0.0)	-
Unknown/not reported	-	11 (9.2)	-
Other	-	18 (15.0)	-
Age, y (range)	64.7 (22-94)	60.5 (22-87)	62.6 (22-94)
BMI, n (%)			
<25	56 (46.7)	41 (34.2)	97 (40.4)
≤25-30	44 (36.7)	49 (40.8)	93 (38.8)
≥30	20 (16.7)	30 (25.0)	50 (20.8)
Prior cardiac diagnoses, n (%)			
Hypertension	34 (28.3)	66 (55.0)	100 (41.7)
Hyperlipidemia	26 (21.7)	70 (58.3)	96 (40.0)
Diabetes	19 (15.8)	23 (19.2)	42 (17.5)
Heart failure	8 (6.7)	14 (11.7)	22 (9.2)
Atrial fibrillation	25 (20.8)	10 (8.3)	35 (14.6)
Other arrhythmias	9 (7.5)	9 (7.5)	18 (7.5)
Coronary artery disease	30 (25.0)	19 (15.8)	49 (20.4)
Prior heart attack	14 (11.7)	0 (0.0)	14 (5.8)
Valvular heart disease	31 (25.8)	11 (9.2)	42 (17.5)
Pulmonary hypertension	4 (3.3)	3 (2.5)	7 (2.9)
Heart transplant	4 (3.3)	1 (0.8)	5 (2.1)
Cardiomyopathies	18 (15.0)	7 (5.8)	25 (10.4)
Congenital heart disease	0 (0.0)	1 (0.8)	1 (0.4)
Other	5 (4.2)	12 (10.0)	17 (7.1)
None	21 (17.5)	21 (17.5)	42 (17.5)
Not reported	1 (0.8)	1 (0.8)	2 (0.8)
Prior noncardiac diagnoses, n (%)			
Renal disease	11 (9.2)	12 (10.0)	23 (9.6)
COPD/emphysema	6 (5.0)	5 (4.2)	11 (4.6)
Pulmonary embolus	2 (1.7)	5 (4.2)	7 (2.9)
Systemic infiltrative disease	0 (0.0)	1 (0.8)	1 (0.4)
Cancer	22 (18.3)	26 (21.7)	48 (20.0)
Underwent chemotherapy	2 (1.7)	12 (10.0)	14 (5.8)
Underwent radiation	3 (2.5)	3 (2.5)	6 (2.5)
Other	27 (22.5)	46 (38.3)	73 (30.4)
None	58 (48.3)	44 (36.7)	102 (42.5)
Not reported	1 (0.8)	3 (2.5)	4 (1.7)

BMI = body mass index; COPD = chronic obstructive pulmonary disease; FAS = full analysis set.

For primary endpoints, 100% of the exams performed by novices using the studied software met the quality standard for visual analysis of the LV size and function, the RV size, and the presence or absence of nontrivial PE. Furthermore, there was no difference in these primary endpoints between novice and expert exams (see Table 2). Given the 100% quality rate, no differences could be highlighted across various subgroup analyses (by investigator center, known cardiac abnormality, and BMI).

**Table 2:** Difference Novice-Expert: Primary and Secondary Endpoints

	Novice Scans of Sufficient Quality	Expert Scans of Sufficient Quality	Novice-Expert Difference % Points
Primary endpoints			
QVA of LV size	240 (100) [98.4; 100]	240 (100) [98.4; 100]	0.0
QVA of global LV function	240 (100) [98.4; 100]	240 (100) [98.4; 100]	0.0
QVA of RV size	240 (100) [98.4; 100]	240 (100) [98.4; 100]	0.0
QVA of nontrivial PE	240 (100) [98.4; 100]	240 (100) [98.4; 100]	0.0
Secondary endpoints			
QVA of RV function	239 (99.6) [97.7; 99.9]	240 (100) [98.4; 100]	−0.4
QVA of left atrial size	240 (100) [98.4; 100]	240 (100) [98.4; 100]	0.0
QVA of right atrial size	237 (98.8) [96.4; 99.6]	240 (100) [98.4; 100]	−1.2
QVA of LV segmental kinetics	229 (95.4) [92.0; 97.4]	240 (100) [98.4; 100]	−4.6
QVA of aortic valve	237 (98.8) [96.4; 99.6]	240 (100) [98.4; 100]	−1.2
QVA of mitral valve	240 (100) [98.4; 100]	240 (100) [98.4; 100]	0.0
QVA of tricuspid valve	229 (95.4) [92.0; 97.4]	238 (99.2) [97.0; 99.8]	−3.8
QVA of IVC size	188 (78.3) [72.7; 83.1]	236 (98.3) [95.8; 99.4]	−20.0
DQC for A2C	198 (82.5) [77.2; 86.8]	236 (98.3) [95.8; 99.4]	−15.8
DQC for A3C	216 (90.0) [85.6; 93.2]	240 (100) [98.4; 100]	−10.0
DQC for A4C	231 (96.2) [93.0; 98.0]	240 (100) [98.4; 100]	−3.8
DQC for A5C	224 (93.3) [89.4; 95.9]	238 (99.2) [97.0; 99.8]	−5.8
DQC for PLAX	234 (97.5) [94.7; 98.8]	240 (100) [98.4; 100]	−2.5
DQC for PSAX at the aortic valve	214 (89.2) [84.6; 92.5]	238 (99.2) [97.0; 99.8]	−10.0
DQC for PSAX at the mitral valve	218 (90.8) [86.5; 93.9]	239 (99.6) [97.7; 99.9]	−8.8
DQC for PSAX at the papillary muscles	233 (97.1) [94.1; 98.6]	238 (99.2) [97.0; 99.8]	−2.1
DQC for subcostal 4-chamber	214 (89.2) [84.6; 92.5]	236 (98.3) [95.8; 99.4]	−9.2
DQC for SC-IVC	186 (77.5) [71.8; 82.3]	236 (98.3) [95.8; 99.4]	−20.8

Values are n (%) [95% Wilson CI] unless otherwise indicated. Percentages are based on all patients from the FAS population examined by a novice and an expert with 10 reviews, excluding those with missing values. CI presented is 2-sided.

Results obtained for the achievement of QVA and the DQC measurement.

A2C = apical 2-chamber; A3C = apical 3-chamber; A4C = apical 4-chamber; A5C = apical 5-chamber; DQC = diagnostic quality clip; FAS = full analysis set; IVC = inferior vena cava; LV = left ventricle; PE = pericardial effusion; PLAX = parasternal long-axis; PSAX = parasternal short-axis; QVA = qualitative visual assessment; RV = right ventricle; SC-IVC = subcostal inferior vena cava.

For nearly all secondary clinical parameters (7 out of 8), including RV function, left and right atrial size, LV segmental wall motion, and aortic/mitral/tricuspid valves, over 95% (ranging from 95.4% to 100%) of the exams conducted by novices with guidance from the studied device were of sufficient quality for qualitative visual assessment (see Table 2). For the IVC size (the eighth parameter), 78.3% of these exams were of sufficient quality for qualitative visual assessment. In terms of diagnostic quality assessment of clips, the difference between novice and expert exams ranged from 2% for the PSAX-PM view to 20.8% for the subcostal IVC view. No significant differences between the French and the American centers, nor for BMI groups (see Table 3), nor for patients with and without cardiac abnormality (see Supplemental Tables 2 and 3), were established. The overall agreement among cardiologists in categorizing 12 clinical key parameters as normal/borderline or abnormal was >85% (ranging from 87.1% to 99.6%) for exams performed by novices (see Supplemental Table 4). The analysis of measurements obtained by the panel of 5 expert cardiologists revealed strong (Pearson correlation coefficient >0.60) or very strong correlations (>0.80) between measurements obtained from expert acquisitions and those from novice acquisitions. In addition, the interobserver study results show strong agreement between the expert cardiologists on both novice and expert exams. This consistency is a strong indicator of interobserver reliability, suggesting that the expert cardiologists were intuitively aligned among themselves.

**Table 3:** Performance of Novice Scans by BMI: Primary and Secondary Endpoints

	BMI <25 (n = 97)	BMI 25 to <30 (n = 93)	BMI ≥30 (n = 50)
Primary endpoints			
QVA of LV size	97 (100) [96.2; 100]	93 (100) [96.0; 100]	50 (100) [92.9; 100]
QVA of global LV function	97 (100) [96.2; 100]	93 (100) [96.0; 100]	50 (100) [92.9; 100]
QVA of RV size	97 (100) [96.2; 100]	93 (100) [96.0; 100]	50 (100) [92.9; 100]
QVA of nontrivial PE	97 (100) [96.2; 100]	93 (100) [96.0; 100]	50 (100) [92.9; 100]
Secondary endpoints			
QVA of RV function	97 (100) [96.2; 100]	93 (100) [96.0; 100]	49 (98.0) [89.5; 99.6]
QVA of left atrial size	97 (100) [96.2; 100]	93 (100) [96.0; 100]	50 (100) [92.9; 100]
QVA of right atrial size	97 (100) [96.2; 100]	91 (97.8) [92.5; 99.4]	49 (98.0) [89.5; 99.6]
QVA of LV segmental kinetics	93 (95.9) [89.9; 98.4]	90 (96.8) [90.9; 98.9]	46 (92.0) [81.2; 96.8]
QVA of aortic valve	95 (97.9) [92.8; 99.4]	93 (100) [96.0; 100]	49 (98.0) [89.5; 99.6]
QVA of mitral valve	97 (100) [96.2; 100]	93 (100) [96.0; 100]	50 (100) [92.9; 100]
QVA of tricuspid valve	95 (97.9) [92.8; 99.4]	88 (94.6) [88.0; 97.7]	46 (92.0) [81.2; 96.8]
QVA of IVC size	83 (85.6) [77.2; 91.2]	68 (73.1) [63.3; 81.1]	37 (74.0) [60.4; 84.1]
DQC for A2C	84 (86.6) [78.4; 92.0]	77 (82.8) [73.9; 89.1]	37 (74.0) [60.4; 84.1]
DQC for A3C	87 (89.7) [82.1; 94.3]	84 (90.3) [82.6; 94.8]	45 (90.0) [78.6; 95.7]
DQC for A4C	95 (97.9) [92.8; 99.4]	90 (96.8) [90.9; 98.9]	46 (92.0) [81.2; 96.8]
DQC for A5C	91 (93.8) [87.2; 97.1]	89 (95.7) [89.5; 98.3]	44 (88.0) [76.2; 94.4]
DQC for PLAX	93 (95.9) [89.9; 98.4]	92 (98.9) [94.2; 99.8]	49 (98.0) [89.5; 99.6]
DQC for PSAX at the aortic valve	86 (88.7) [80.8; 93.5]	85 (91.4) [83.9; 95.6]	43 (86.0) [73.8; 93.0]
DQC for PSAX at the mitral valve	92 (94.8) [88.5; 97.8]	85 (91.4) [83.9; 95.6]	41 (82.0) [69.2; 90.2]
DQC for PSAX at the papillary muscles	94 (96.9) [91.3; 98.9]	92 (98.9) [94.2; 99.8]	47 (94.0) [83.8; 97.9]
DQC for subcostal 4-chamber	91 (93.8) [87.2; 97.1]	81 (87.1) [78.8; 92.5]	42 (84.0) [71.5; 91.7]
DQC for SC-IVC	82 (84.5) [76.0; 90.4]	67 (72.0) [62.2; 80.1]	37 (74.0) [60.4; 84.1]

Values are n (%) [95% Wilson CI]. Percentages are based on all patients from FAS population examined by a novice with 5 reviews, excluding those with missing values. CI presented is 2-sided.

Results obtained for the achievement of QVA and the DQC measurement for novice scans by BMI (BMI <25 kg/m^2^ [normal], 25 ≤ BMI <30 kg/m^2^ [overweight], BMI ≥30 kg/m^2^ [obese]).

Abbreviations as in Tables 1 and 2.

The objective for novices was to acquire 10 clips per patient, for a total of 2,400 clips. Novices recorded 2,362 clips, missing 38 clips (when novices were unable to record a reference view). Of the recorded clips, 67.4% were saved using the “auto record” feature, 21.3% with the “best-effort record” feature, and 11.3% with the “manual record” feature. Diagnostic quality was achieved on 99.7% of the clips recorded with the “auto record” feature, 93.9% of the clips recorded with the “best-effort record” feature, and 40.5% of the clips recorded with the “manual record.”

Although acquisition times were only recorded for novices (mean 23.6 ± 10.6 minutes), expert-acquired exams typically require less time, though not formally measured here. When comparing novice performance over time (ie, across exams 0-10, 11-20, and 21-30), high percentages of diagnostic quality exams were achieved already at the beginning of the trial (see Supplemental Table 5).

## Discussion

This international prospective clinical trial demonstrated that novices can effectively perform heart exams for the assessment of the LV size and function, the RV size, and the presence of a nontrivial PE with the studied AI-based software. The agreement between the acquisitions obtained by the experts (sonographers/cardiologists) and those obtained by the novice nurses was notably high, both in terms of acquisition quality and echocardiographic parameters. These results were consistent across both investigating centers from different countries. The presence of cardiac abnormalities or obesity did not impact the quality of the heart exam. No adverse event, side effect, or device deficiency was observed during this clinical trial with the evaluated device.

This clinical trial demonstrated that novices can perform heart exams, allowing the evaluation of the most important cardiac parameters. In both countries, novices had medical training of at least 2 years. No differences were observed in the performance for the primary endpoints between the French and the American centers despite differences in demographics across the 2 sites. There were more men included in France than in the United States (60.8% vs 41.7%), fewer obese patients (BMI ≥30: 16.7% vs 25%), and more cardiac abnormalities (at least 1 cardiac abnormality: 75% vs 65.8%). No significant differences were observed between both centers.

Traditional training for health care professionals in the acquisition and interpretation of cardiac ultrasound takes several years. In the United States, achieving level 3 certification—the highest standard in echocardiography—requires 9 months of specialized fellowship training, as outlined by the American College of Cardiology and the American Society of Echocardiography. In France, cardiologists pursue a 2-year university diploma in echocardiography in addition to their core cardiology training. Similarly, all sonographers complete at least 2 years of formal education to be certified.

The use of AI-based technology, like the HeartFocus software studied in this trial, could enable any health care professional to perform point-of-care ultrasound exams after a brief training.

To our knowledge, 2 existing AI technologies designed to guide image acquisition have been evaluated prospectively with novice users.^14^^,^^15^ These teams have conducted a prospective multicenter clinical study with the same clinical design, number of patients, and primary objectives. Compared to these solutions, our study evaluates a different AI guidance system, includes both European and American sites, and assesses a broader set of cardiac structures (eg, right atrium size and LV segmental wall motion). The software evaluated in our trial achieved comparable or higher rates of diagnostic-quality scans for several endpoints (ie, while Narang et al showed 92.5% diagnostic-quality RV imaging and Mor-Avi et al showed 93%, our study reached 100% for RV size) and over 95% for 7 of 8 secondary endpoints, including in patients with obesity.

Based on the clinical data specific to the studied software and information on similar devices, we have demonstrated that it is a safe and reliable device to use in clinical practice. It provides a means for novices to acquire cardiac ultrasound exams after a short training.

On a larger scale, AI software such as HeartFocus has the potential to unlock access to ultrasound exams by allowing nonexperts (emergency physicians, residents, general practitioners, primary care physicians, nurses, etc) to acquire cardiac ultrasound exams of sufficient quality for clinical diagnosis and decision-making. Such software could spread access to cardiac ultrasound in rural medical practices, rehabilitation, and skilled nursing facilities, or anywhere else where sonographers and cardiologists are in shortage. Future research studies may be considered to demonstrate the benefit for patients in these different settings. Other research opportunities may seek to expand this technology to be used as a screening tool for certain diagnostic conditions or to perform limited follow-up exams once patients have been discharged from a medical center with full echocardiography services.

### Study Limitations

Several limitations should be acknowledged in this clinical study. Patient recruitment was restricted to specific settings, excluding intensive care units or emergency departments. Additionally, not all health care environments were represented in this clinical study conducted in 2 large teaching hospitals in the United States and France. Although our study did not include a control group of novices scanning without AI guidance, prior literature^20^^,^^21^ suggests that unassisted novices typically require more extensive training and achieve lower image quality. Including a crossover group would be a valuable next step to quantify the incremental value of AI guidance. Furthermore, the studied software was only tested with a specific hand-held probe; in clinical practice, it could be used with other ultrasound systems. While the device provides guidance on acquiring 10 cardiac ultrasound views, clinical interpretation remains the responsibility of a cardiologist.

Perspectives **COMPETENCY IN MEDICAL KNOWLEDGE:** This study demonstrates that novice health care providers, specifically nurses without prior ultrasound experience, can acquire diagnostic-quality transthoracic echocardiographic images using an AI-guided system. The results showed high agreement with expert acquisitions for key clinical parameters, including LV and RV size and function, with only minimal training. This supports the growing role of AI in enabling broader clinical use of cardiac imaging. The integration of AI guidance into ultrasound workflows may allow health systems to extend echocardiography access beyond specialized cardiology services. With increasing imaging demands and workforce shortages, particularly in underserved or rural areas, AI-based solutions can help distribute diagnostic capacity across more providers. These tools can reduce wait times and facilitate earlier clinical decision-making by empowering frontline staff to perform high-quality scans.

**TRANSLATIONAL OUTLOOK:** AI-guided ultrasound acquisition has the potential to transform how cardiac imaging is delivered across health care settings. Future studies should assess this technology's clinical and operational impact in emergency departments, primary care, and remote or resource-limited environments. Additionally, health-economic evaluations will be essential to inform reimbursement models and integration into national care pathways.

## Funding support and author disclosures

This work was supported by DESKi, a French medtech company, developing the HeartFocus software as a medical device to assist medical professionals in the acquisition of cardiac ultrasound images. DESKi won a funding grant for medical imaging projects from the French government (AAP France 2030) to help the company carry out this clinical study to validate the safety and performance of the HeartFocus medical device. Drs Rodrigues, Roger, B. Moal, and O. Moal are employees of DESKi. Drs B. Moal and O. Moal are co-founders of DESKi and have shared ownership of DESKi. All other authors have reported that they have no relationships relevant to the contents of this paper to disclose.
