# Supplementary material for: Artificial Intelligence Empowers Novice Users to Acquire Diagnostic-Quality Echocardiography
Source: JACC Adv. 2025 Jul 22;4(8):102005. doi: 10.1016/j.jacadv.2025.102005 (PMC12308015; doi:10.1016/j.jacadv.2025.102005)
Supplement: Supplementary data [file mmc1.pdf]

<u>Supplemental Content</u>

Supplementary Table 1. Presence of Cardiac abnormalities in the Full Analysis Set (FAS) population (N=240).

Supplementary Table 2. Performance of novice scans by center - primary and secondary endpoints. Results obtained for the achievement of Qualitative Visual Assessment (QVA) and the Diagnostic Quality Clip (DQC) measurement for novices' scans, by center (N=120), and in the Full Analysis Set (FAS) population (N=240).

Supplementary Table 3. Performance of novice scans by cardiac abnormality - primary and secondary endpoints. Results obtained for the achievement of Qualitative Visual Assessment (QVA) and the Diagnostic Quality Clip (DQC) measurement for novice scans, by cardiac abnormality (known or none) and in the Full Analysis Set (FAS) population (N=240).

Supplementary Table 4. Cross-Classification of Cardiologists' Clinical Assessment Using Novice Acquired vs. Expert Acquired exams - Primary and Secondary Endpoints Qualitative Visual Assessment among Patients with both scans, allowing the Qualitative Visual Assessment (QVA)

Supplementary Table 5. Performance of novice scans by exam sequence number (ESN) - primary and secondary endpoints. Results obtained for the achievement of Qualitative Visual Assessment (QVA) and the Diagnostic Quality Clip (DQC) measurement for novice scans, by exam sequence number (the first 10 exams, 11 to 21 exams, and the last 9 exams) and in the Full Analysis Set (FAS) population (N=240).

The authors have provided this supplemental material to give readers additional information about their work.

Supplementary Table 1. Presence of Cardiac abnormalities in the Full Analysis Set (FAS) population (N=240).

	France (N=120)	***United States (N=120)***	***FAS population (N=240)***
Presence of at least one cardiac abnormality, N (%)	*120*	*120*	*240*
Yes	*90 (75.0)*	*79 (65.8)*	*169 (70.4)*
No	*30 (25.0)*	*41 (34.2)*	*71 (29.6)*
Not reported	*0 (0.0)*	*0 (0.0)*	*0 (0.0)*
Missing	*0*	*0*	*0*
Cardiac abnormalities [a], N (%)	*120*	*120*	*240*
Abnormal left ventricular size or function	*46 (38.3)*	*35 (29.2)*	*81 (33.8)*
Abnormal aortic valve	*45 (37.5)*	*19 (15.8)*	*64 (26.7)*
Abnormal left atrial size	*33 (27.5)*	*20 (16.7)*	*53 (22.1)*
Abnormal mitral valve	*31 (25.8)*	*17 (14.2)*	*48 (20.0)*
Abnormal right atrial size	*21 (17.5)*	*9 (7.5)*	*30 (12.5)*
Abnormal tricuspid valve	*18 (15.0)*	*9 (7.5)*	*27 (11.2)*
Abnormal right ventricular size or function	*16 (13.3)*	*4 (3.3)*	*20 (8.3)*
Non-trivial pericardial effusion	*5 (4.2)*	*5 (4.2)*	*10 (4.2)*
Septal defect	*7 (5.8)*	*3 (2.5)*	*10 (4.2)*
Abnormal inferior vena cava size	*9 (7.5)*	*0 (0.0)*	*9 (3.8)*
Patent foramen oval	*0 (0.0)*	*1 (0.8)*	*1 (0.4)*
Other	*9 (7.5)*	*30 (25.0)*	*39 (16.2)*

[a] One patient may have several cardiac abnormalities.
Percentages are based on all patients from the FAS population, excluding those with missing values.

Supplementary Table 2. Performance of novice scans by center - primary and secondary endpoints. Results obtained for the achievement of Qualitative Visual Assessment (QVA) and the Diagnostic Quality Clip (DQC) measurement for novice scans, by center (N=120).

Clinical parameter	***France (N=120) n (%) [95% Wilson CI]***	***United States (N=120) n (%) [95% Wilson CI]***
Primary endpoints		
QVA of LV size	*120 (100) [96.9;100]*	*120 (100) [96.9;100]*
QVA of global LV function	*120 (100) [96.9;100]*	*120 (100) [96.9;100]*
QVA of RV size	*120 (100) [96.9;100]*	*120 (100) [96.9;100]*
QVA of non-trivial PE	*120 (100) [96.9;100]*	*120 (100) [96.9;100]*
Secondary endpoints		
QVA of RV function	*120 (100) [96.9;100]*	*119 (99.2) [95.4;99.9]*
QVA of left atrial size	*120 (100) [96.9;100]*	*120 (100) [96.9;100]*
QVA of right atrial size	*118 (98.3) [94.1;99.5]*	*119 (99.2) [95.4;99.9]*
QVA of LV segmental kinetics	*113 (94.2) [88.4;97.1]*	*116 (96.7) [91.7;98.7]*
QVA of aortic valve	*118 (98.3) [94.1;99.5]*	*119 (99.2) [95.4;99.9]*
QVA of mitral valve	*120 (100) [96.9;100]*	*120 (100) [96.9;100]*
QVA of tricuspid valve	*111 (92.5) [86.4;96.0]*	*118 (98.3) [94.1;99.5]*
QVA of IVC size	*90 (75.0) [66.6;81.9]*	*98 (81.7) [73.8;87.6]*
DQC for A2C	*92 (76.7) [68.3;83.3]*	*106 (88.3) [81.4;92.9]*
DQC for A3C	*106 (88.3) [81.4;92.9]*	*110 (91.7) [85.3;95.4]*
DQC for A4C	*114 (95.0) [89.5;97.7]*	*117 (97.5) [92.9;99.1]*
DQC for A5C	*111 (92.5) [86.4;96.0]*	*113 (94.2) [88.4;97.1]*
DQC for PLAX	*116 (96.7) [91.7;98.7]*	*118 (98.3) [94.1;99.5]*
DQC for PSAX at the aortic valve	*104 (86.7) [79.4;91.6]*	*110 (91.7) [85.3;95.4]*
DQC for PSAX at the mitral valve	*109 (90.8) [84.3;94.8]*	*109 (90.8) [84.3;94.8]*
DQC for PSAX at the papillary muscles	*116 (96.7) [91.7;98.7]*	*117 (97.5) [92.9;99.1]*
DQC for subcostal 4-chamber	*104 (86.7) [79.4;91.6]*	*110 (91.7) [85.3;95.4]*
DQC for SC-IVC	*89 (74.2) [65.7;81.2]*	*97 (80.8) [72.9;86.9]*

Percentages are based on all patients from FAS population (N=240) examined by a novice with 5 reviews, excluding those with missing values. CI presented is 2-sided.

Supplementary Table 3. Performance of novice scans by cardiac abnormality - primary and secondary endpoints. Results obtained for the achievement of Qualitative Visual Assessment (QVA) and the Diagnostic Quality Clip (DQC) measurement for novice scans, by cardiac abnormality (known or none).

Clinical parameter	***Known cardiac abnormality at the time of enrollment (N=169) n (%) [95% Wilson CI]***	***No known cardiac abnormality at the time of enrollment (N=71) n (%) [95% Wilson CI]***
Primary endpoints		
QVA of LV size	*169 (100) [97.8;100]*	*71 (100) [94.9;100]*
QVA of global LV function	*169 (100) [97.8;100]*	*71 (100) [94.9;100]*
QVA of RV size	*169 (100) [97.8;100]*	*71 (100) [94.9;100]*
QVA of non-trivial PE	*169 (100) [97.8;100]*	*71 (100) [94.9;100]*
Secondary endpoints		
QVA of RV function	*168 (99.4) [96.7;99.9]*	*71 (100) [94.9;100]*
QVA of left atrial size	*169 (100) [97.8;100]*	*71 (100) [94.9;100]*
QVA of right atrial size	*167 (98.8) [95.8;99.7]*	*70 (98.6) [92.4;99.8]*
QVA of LV segmental kinetics	*160 (94.7) [90.2;97.2]*	*69 (97.2) [90.3;99.2]*
QVA of aortic valve	*168 (99.4) [96.7;99.9]*	*69 (97.2) [90.3;99.2]*
QVA of mitral valve	*169 (100) [97.8;100]*	*71 (100) [94.9;100]*
QVA of tricuspid valve	*159 (94.1) [89.5;96.8]*	*70 (98.6) [92.4;99.8]*
QVA of IVC size	*127 (75.1) [68.1;81.1]*	*61 (85.9) [76.0;92.2]*
DQC for A2C	*137 (81.1) [74.5;86.3]*	*61 (85.9) [76.0;92.2]*
DQC for A3C	*149 (88.2) [82.4;92.2]*	*67 (94.4) [86.4;97.8]*
DQC for A4C	*163 (96.4) [92.5;98.4]*	*68 (95.8) [88.3;98.6]*
DQC for A5C	*158 (93.5) [88.7;96.3]*	*66 (93.0) [84.6;97.0]*
DQC for PLAX	*167 (98.8) [95.8;99.7]*	*67 (94.4) [86.4;97.8]*
DQC for PSAX at the aortic valve	*148 (87.6) [81.8;91.7]*	*66 (93.0) [84.6;97.0]*
DQC for PSAX at the mitral valve	*151 (89.3) [83.8;93.2]*	*67 (94.4) [86.4;97.8]*
DQC for PSAX at the papillary muscles	*164 (97.0) [93.3;98.7]*	*69 (97.2) [90.3;99.2]*
DQC for subcostal 4-chamber	*147 (87.0) [81.1;91.2]*	*67 (94.4) [86.4;97.8]*
DQC for SC-IVC	*125 (74.0) [66.9;80.0]*	*61 (85.9) [76.0;92.2]*

Percentages are based on all patients from FAS population (N=240) examined by a novice with 5 reviews, excluding those with missing values. CI presented is 2-sided.

Supplementary Table 4. Cross-Classification of Cardiologists' Clinical Assessment Using Novice Acquired vs. Expert Acquired exams - Primary and Secondary Endpoints Qualitative Visual Assessment among Patients with both scans, allowing the Qualitative Visual Assessment (QVA)

	**Expert exam**						
**Clinical parameter**<br>**Novice exam**							% Agreement and CI
1. Qualitative Visual Assessment of Left Ventricular size	Normal or borderline	Hypertrophy	Dilation	Hypertrophy and dilation	No majority assessment among cardiologists	Total	
Normal or borderline	*185*	*9*	*4*	*0*	*0*	*198*	*93.4 [90.0; 96.9]*
Hypertrophy	*10*	*17*	*0*	*0*	*0*	*27*	*63.0 [44.7; 81.2]*
Dilation	*2*	*0*	*8*	*0*	*0*	*10*	*80.0 [55.2; 100]*
Hypertrophy and dilation	*0*	*0*	*0*	*0*	*0*	*0*	*NA*
No majority assessment among cardiologists	*3*	*0*	*2*	*0*	*0*	*5*	*0.0 [0.0; 0.0]*
Total	*200*	*26*	*14*	*0*	*0*	*240*	*87.5% [83.3; 91.7]*
2. Qualitative Visual Assessment of Global Left Ventricular function	Normal or borderline		Reduced (EF ≤ 50%)		No majority assessment among cardiologists	Total	
Normal or borderline	*197*		*8*		*0*	*205*	*96.1 [93.4; 98.7]*
Reduced (EF ≤ 50%)	*3*		*32*		*0*	*35*	*91.4 [82.2; 100]*
No majority assessment	*0*		*0*		*0*	*0*	*NA*
Total	*200*		*40*		*0*	*240*	*95.4% [92.8; 98.1]*
3. Qualitative Visual Assessment of Right Ventricular size	Normal or borderline	Hypertrophy	Dilation	Hypertrophy and dilation	No majority assessment among cardiologists	Total	
Normal or borderline	*228*	*0*	*1*	*0*	*0*	*229*	*99.6 [98.7; 100]*
Hypertrophy	*0*	*0*	*0*	*0*	*0*	*0*	*NA*
Dilation	*4*	*0*	*6*	*0*	*0*	*10*	*60.0 [29.6; 90.4]*
Hypertrophy and dilation	*0*	*0*	*0*	*0*	*0*	*0*	*NA*
No majority assessment among cardiologists	*1*	*0*	*0*	*0*	*0*	*1*	*0.0 [0.0; 0.0]*
Total	*233*	*0*	*7*	*0*	*0*	*240*	*97.5% [95.5; 99.5]*
4. Qualitative Visual Assessment of non-trivial Pericardial Effusion	Absent		Present		No majority assessment among cardiologists	Total	
Absent	*233*		*2*		*0*	*235*	*99.1 [98.0; 100]*
Present	*2*		*3*		*0*	*5*	*60.0 [17.1; 100]*
No majority assessment among cardiologists	*0*		*0*		*0*	*0*	*NA*
Total	*235*		*5*		*0*	*240*	*98.3% [96.7; 100.0]*
5. Qualitative Visual Assessment of the Right Ventricular function	Normal or borderline		Reduced		No majority assessment among cardiologists	Total	
Normal or borderline	*228*		*1*		*0*	*229*	*99.6 [98.7; 100]*
Reduced	*3*		*5*		*0*	*8*	*62.5 [29.0; 96.0]*
No majority assessment among cardiologists	*2*		*0*		*0*	*2*	*0.0 [0.0; 0.0]*
Total	*233*		*6*		*0*	*239*	*97.5% [95.5; 99.5]*
6. Qualitative Visual Assessment of the Left Atrial size	Normal or borderline		Abnormal (enlarged)		No majority assessment among cardiologists	Total	
Normal or borderline	*189*		*22*		*1*	*212*	*89.2 [85.0; 93.3]*

Abnormal (enlarged)	*7*		*20*	*0*	*27*	*74.1 [57.5; 90.6]*
No majority assessment among cardiologists	*0*		*1*	*0*	*1*	*0.0 [0.0; 0.0]*
Total	*196*		*43*	*1*	*240*	*87.1% [82.8; 91.3]*
7. Qualitative Visual Assessment of the Right Atrial size	Normal or borderline		Abnormal (enlarged)	No majority assessment among cardiologists	Total	
Normal or borderline	*208*		*7*	*0*	*215*	*96.7 [94.4; 99.1]*
Abnormal (enlarged)	*10*		*11*	*0*	*21*	*52.4 [31.0; 73.7]*
No majority assessment among cardiologists	*0*		*1*	*0*	*1*	*0.0 [0.0; 0.0]*
Total	*218*		*19*	*0*	*237*	*92.4% [89.0; 95.8]*
8. Qualitative Visual Assessment of the Left Ventricular segmental kinetics	Normal or borderline		Abnormal	No majority assessment among cardiologists	Total	
Normal or borderline	*187*		*5*	*0*	*192*	*97.4 [95.1; 99.6]*
Abnormal	*9*		*25*	*1*	*35*	*71.4 [56.5; 86.4]*
No majority assessment among cardiologists	*2*		*0*	*0*	*2*	*0.0 [0.0; 0.0]*
Total	*198*		*30*	*1*	*229*	*92.6% [89.2; 96.0]*
9. Qualitative Visual Assessment of the Aortic Valve	Structurally normal	Structurally abnormal	Suspected device	No majority assessment among cardiologists	Total	
Structurally normal	*180*	*9*	*2*	*0*	*191*	*94.2 [90.9; 97.5]*
Structurally abnormal	*6*	*22*	*2*	*0*	*30*	*73.3 [57.5; 89.2]*
Suspected device	*0*	*1*	*15*	*0*	*16*	*93.8 [81.9; 100]*
No majority assessment among cardiologists	*0*	*0*	*0*	*0*	*0*	*NA*
Total	*186*	*32*	*19*	*0*	*237*	*91.6% [88.0; 95.1]*
10. Qualitative Visual Assessment of the Mitral Valve	Structurally normal	Structurally abnormal	Suspected device	No majority assessment among cardiologists	Total	
Structurally normal	*216*	*1*	*1*	*0*	*218*	*99.1 [97.8; 100]*
Structurally abnormal	*2*	*8*	*1*	*0*	*11*	*72.7 [46.4; 99.0]*
Suspected device	*1*	*0*	*8*	*1*	*10*	*80.0 [55.2; 100]*
No majority assessment among cardiologists	*1*	*0*	*0*	*0*	*1*	*0.0 [0.0; 0.0]*
Total	*220*	*9*	*10*	*1*	*240*	*96.7% [94.4; 98.9]*
11. Qualitative Visual Assessment of the Tricuspid Valve	Structurally normal	Structurally abnormal	Suspected device	No majority assessment among cardiologists	Total	
Structurally normal	*225*	*0*	*0*	*0*	*225*	*100 [100; 100]*
Structurally abnormal	*0*	*0*	*0*	*0*	*0*	*NA*
Suspected device	*0*	*0*	*2*	*0*	*2*	*100 [100; 100]*
No majority assessment among cardiologists	*0*	*0*	*1*	*0*	*1*	*0.0 [0.0; 0.0]*
Total	*225*	*0*	*3*	*0*	*228*	*99.6% [98.7; 100]*
12. Qualitative Visual Assessment of the Inferior Vena Cava size	Normal	Dilated	No majority assessment among cardiologists		Total	
Normal	*158*	*10*	*0*		*168*	*94.0 [90.5; 97.6]*
Dilated	*11*	*8*	*0*		*19*	*42.1 [19.9; 64.3]*
No majority assessment among cardiologists	*0*	*0*	*0*		*0*	*NA*
Total	*169*	*18*	*0*		*187*	*88.8% [84.2; 93.3]*

Percentages are based on all patients from Full Analysis Set (FAS) population examined by a novice and an expert with both scans allowing the QVA of several clinical parameters excluding those with missing values. CI presented are 2-sided.

Supplementary Table 5. Performance of novice scans by exam sequence number (ESN) - primary and secondary endpoints. Results obtained for the achievement of Qualitative Visual Assessment (QVA) and the Diagnostic Quality Clip (DQC) measurement for novice scans, by exam sequence number (the first 10 exams, 11 to 21 exams, and the last 9 exams) and in the Full Analysis Set (FAS) population (N=240).

Clinical parameter	***ESN < 11 (N=80) n (%) [95% Wilson CI]***	***ESN 11 to < 21 (N=80) n (%) [95% Wilson CI]***	***ESN ≥ 21 (N=80) n (%) [95% Wilson CI]***	***FAS population (N=240) n (%) [95% Wilson CI]***
Primary endpoints				
QVA of LV size	*80 (100) [95.4;100]*	*80 (100) [95.4;100]*	*80 (100) [95.4;100]*	*240 (100) [98.4;100]*
QVA of global LV function	*80 (100) [95.4;100]*	*80 (100) [95.4;100]*	*80 (100) [95.4;100]*	*240 (100) [98.4;100]*
QVA of RV size	*80 (100) [95.4;100]*	*80 (100) [95.4;100]*	*80 (100) [95.4;100]*	*240 (100) [98.4;100]*
QVA of non-trivial PE	*80 (100) [95.4;100]*	*80 (100) [95.4;100]*	*80 (100) [95.4;100]*	*240 (100) [98.4;100]*
Secondary endpoints				
QVA of RV function	*79 (98.8) [93.3;99.8]*	*80 (100) [95.4;100]*	*80 (100) [95.4;100]*	*239 (99.6) [97.7;99.9]*
QVA of left atrial size	*80 (100) [95.4;100]*	*80 (100) [95.4;100]*	*80 (100) [95.4;100]*	*240 (100) [98.4;100]*
QVA of right atrial size	*78 (97.5) [91.3;99.3]*	*79 (98.8) [93.3;99.8]*	*80 (100) [95.4;100]*	*237 (98.8) [96.4;99.6]*
QVA of LV segmental kinetics	*77 (96.2) [89.5;98.7]*	*77 (96.2) [89.5;98.7]*	*75 (93.8) [86.2;97.3]*	*229 (95.4) [92.0;97.4]*
QVA of aortic valve	*78 (97.5) [91.3;99.3]*	*80 (100) [95.4;100]*	*79 (98.8) [93.3;99.8]*	*237 (98.8) [96.4;99.6]*
QVA of mitral valve	*80 (100) [95.4;100]*	*80 (100) [95.4;100]*	*80 (100) [95.4;100]*	*240 (100) [98.4;100]*
QVA of tricuspid valve	*76 (95.0) [87.8;98.0]*	*77 (96.2) [89.5;98.7]*	*76 (95.0) [87.8;98.0]*	*229 (95.4) [92.0;97.4]*
QVA of IVC size	*62 (77.5) [67.2;85.3]*	*65 (81.2) [71.3;88.3]*	*61 (76.2) [65.9;84.2]*	*188 (78.3) [72.7;83.1]*
DQC for A2C	*65 (81.2) [71.3;88.3]*	*65 (81.2) [71.3;88.3]*	*68 (85.0) [75.6;91.2]*	*198 (82.5) [77.2;86.8]*
DQC for A3C	*72 (90.0) [81.5;94.8]*	*74 (92.5) [84.6;96.5]*	*70 (87.5) [78.5;93.1]*	*216 (90.0) [85.6;93.2]*
DQC for A4C	*78 (97.5) [91.3;99.3]*	*77 (96.2) [89.5;98.7]*	*76 (95.0) [87.8;98.0]*	*231 (96.2) [93.0;98.0]*
DQC for A5C	*75 (93.8) [86.2;97.3]*	*74 (92.5) [84.6;96.5]*	*75 (93.8) [86.2;97.3]*	*224 (93.3) [89.4;95.9]*
DQC for PLAX	*78 (97.5) [91.3;99.3]*	*79 (98.8) [93.3;99.8]*	*77 (96.2) [89.5;98.7]*	*234 (97.5) [94.7;98.8]*
DQC for PSAX at the aortic valve	*70 (87.5) [78.5;93.1]*	*71 (88.8) [80.0;94.0]*	*73 (91.2) [83.0;95.7]*	*214 (89.2) [84.6;92.5]*
DQC for PSAX at the mitral valve	*71 (88.8) [80.0;94.0]*	*72 (90.0) [81.5;94.8]*	*75 (93.8) [86.2;97.3]*	*218 (90.8) [86.5;93.9]*
DQC for PSAX at the papillary muscles	*75 (93.8) [86.2;97.3]*	*78 (97.5) [91.3;99.3]*	*80 (100) [95.4;100]*	*233 (97.1) [94.1;98.6]*
DQC for subcostal 4-chamber	*70 (87.5) [78.5;93.1]*	*73 (91.2) [83.0;95.7]*	*71 (88.8) [80.0;94.0]*	*214 (89.2) [84.6;92.5]*
DQC for SC-IVC	*62 (77.5) [67.2;85.3]*	*63 (78.8) [68.6;86.3]*	*61 (76.2) [65.9;84.2]*	*186 (77.5) [71.8;82.3]*

Percentages are based on all patients from FAS population (N=240) examined by a novice with 5 reviews, excluding those with missing values. CI presented is 2-sided.

STARD 2015 Checklist

Section & Topic	No.	Item	✔ (N/A)
**TITLE OR ABSTRACT**			
	1	Identification as a study of diagnostic accuracy using at least one measure of accuracy (such as sensitivity, specificity, predictive values, or AUC)	done, with the proportion of diagnostic quality exams
**ABSTRACT**			
	2	Structured summary of study design, methods, results, and conclusions (for specific guidance, see STARD for Abstracts)	done, with “Study Design” included in “Methods” following the journal recommandations
**INTRODUCTION**			
	3	Scientific & clinical background, including the intended use and clinical role of the index test	done, first paragraphs
	4	Study objectives and hypotheses	done, last paragraph
**METHODS**			
Study design	5	Whether data collection was planned before the index test and reference standard were performed (prospective study) or after (retrospective study)	done, section “Selection and Description of Participants”
Participants	6	Eligibility criteria	done, section “Selection and Description of Participants”
	7	On what basis potentially eligible participants were identified (such as symptoms, results from previous tests, inclusion in registry)	done, section “Selection and Description of Participants”
	8	Where and when potentially eligible participants were identified (setting, location and dates)	done, section “Selection and Description of Participants”
	9	Whether participants formed a consecutive, random or convenience series	done, section “Selection and Description of Participants”
Test methods	10a	Index test, in sufficient detail to allow replication	done, sections “Data Collection” and “Measurements”
	10b	Reference standard, in sufficient detail to allow replication	done, sections “Data Collection” and “Measurements”
	11	Rationale for choosing the reference standard (if alternatives exist)	n/a, we took expert exams as the reference standard and novice exams as the test index
	12a	Definition of and rationale for test positivity cut-offs or result categories of the index test, distinguishing pre-specified from exploratory	n/a, no positive/negative cases
	12b	Definition of and rationale for test positivity cut-offs or result categories of the reference standard, distinguishing pre-specified from exploratory	n/a, no positive/negative cases
	13a	Whether clinical information and reference standard results were available to the performers/readers of the index test	done, section “Measurements”
	13b	Whether clinical information and index test results were available to the assessors of the reference standard	done, section “Measurements”
Analysis	14	Methods for estimating or comparing measures of diagnostic accuracy	done, section “Measurements”
	15	How indeterminate index test or reference standard results were handled	n/a, no indetermination with our study
	16	How missing data on the index test and reference standard were handled	n/a, no missing data with our study
	17	Any analyses of variability in diagnostic accuracy, distinguishing pre-specified from exploratory	done, section “Statistics”
	18	Intended sample size and how it was determined	done, section “Selection and Description of Participants”
**RESULTS**			
Participants	19	Flow of participants, using a diagram. Include the figure number (preferably figure 1) or page number	done, figure 2
	20	Baseline demographic and clinical characteristics of participants	done, table 1
	21a	Distribution of severity of disease in those with the target condition	n/a, no target condition
	21b	Distribution of alternative diagnoses in those without the target condition	n/a, no target condition

	22	Time interval and any clinical interventions between index test and reference standard	n/a, not measured as no impact on the results
Test results	23	Cross tabulation of the index test results (or their distribution) by the results of the reference standard	done, supplementary table 4
	24	Estimates of diagnostic accuracy and their precision (such as 95% confidence intervals)	done, table 2
	25	Any adverse events from performing the index test or the reference standard	done, section “Discussion”
**DISCUSSION**			
	26	Study limitations, including sources of potential bias, statistical uncertainty, and generalisability	done
	27	Implications for practice, including the intended use and clinical role of the index test	done
**OTHER INFORMATION**			
	28	Registration number and name of registry	n/a it is a prospective investigation, submitted to IRB (USA) and Ethics Committee (FR), not a registry
	29	Where the full study protocol can be accessed	done, section “Introduction” NCT number (clinicaltrials.gov)
	30	Sources of funding and other support; role of funders	done, section “Funding” and “Disclosures”

*N/A stands for not applicable and may be a reasonable choice depending on the type of study performed

RSNA®
